# Scavenger Assemblage Behavior at Puma Kills in the Santa Cruz Mountains, California

**DOI:** 10.1002/ece3.73708

**Published:** 2026-05-21

**Authors:** Maximilian L. Allen, Andrew T. L. Allan, Richard M. King, Bethany H. Warner, John J. Morgan, Christopher C. Wilmers

**Affiliations:** ^1^ Illinois Natural History Survey, Prairie Research Institute University of Illinois Champaign Illinois USA; ^2^ Department of Anthropology Durham University Durham UK; ^3^ Center for Integrated Spatial Research, Environmental Studies Department University of California Santa Cruz California USA

**Keywords:** community competition, detection, *Puma concolor*, scavenger, season

## Abstract

Scavengers structure food webs through consuming carrion and cycling nutrients in ecosystems. Scavenger assemblages are shaped by multiple factors, including intra‐ and interspecific competition, environmental conditions, and interactions with apex carnivores. Apex carnivores are particularly important for providing carrion resources to these communities, but can also induce fear effects that affect scavenger spatiotemporal behavior (i.e., landscape of fear). We monitored the scavenger community at 50 carcasses of ungulates killed by pumas ( *Puma concolor* ) in the Santa Cruz Mountains (California, USA), using motion‐activated video camera traps. We hypothesized that obligate avian scavengers (i.e., turkey vultures; *Cathartes aura* ) would visit more kills than opportunistic mammalian scavengers, and that larger mesocarnivores (coyotes [ *Canis latrans* ] and bobcats [ *Lynx rufus* ]) would spend longer durations at carcasses due to competitive dominance. We also hypothesized that seasonal variation in scavenging activity and earlier carcass detection would lead to greater scavenger activity. We found that turkey vultures were the main scavenger, visiting the most carcasses (*n* = 22), most frequently being the first species to visit carcasses (*n* = 14), and spending on average the most time at kills. Larger mesocarnivores had longer mean visit durations, while pumas themselves scavenged from conspecific kills at a relatively high rate. Contrary to our predictions, turkey vultures were the only species with significant seasonal variation—spending more time at kills and finding them more quickly in the dry season. Carcass detection time was weakly associated with the total time a scavenger spent at kills, and scavenger species that discovered more carcasses first were more likely to visit more kills. Our findings highlight the complexity of scavenger assemblages, particularly at kills made by apex carnivores, and contribute to understanding carrion ecology and the role of apex carnivores as ecological brokers in food webs.

## Graphical abstract

The count of the number of kills visited for each scavenger species and the number of kills where the species was the first to discover the carcass.

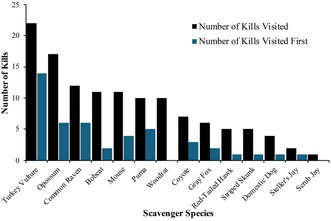

## 1 Introduction

Scavenger communities maintain and stabilize food webs through consuming carrion and cycling nutrients within the ecosystem (Wilson and Wolkovich 2011; Sebastián‐González et al. 2019). The inherent complexity of scavenger communities necessitates the use of multiple metrics to fully understand their composition and activity (Inagaki, Allen, and Koike 2025). Key measures of nutrient and energy cycling include biomass consumption from carcasses and scavenging rates (Ogada et al. 2012; Inagaki et al. 2022; Panda et al. 2023). The time it takes a scavenger to detect a carcass often affects the amount of nutrition available from carrion (Ogada et al. 2012; Inagaki et al. 2022). Interspecific interactions within scavenger communities also provide information on how intraguild competition structures the scavenger community and broader ecosystem (Selva and Fortuna 2007; Prugh and Sivy 2020; Panda et al. 2023). Environmental (e.g., temperature, rainfall) and anthropogenic factors can affect the availability of carrion, ultimately affecting the distribution and assemblage of scavengers that find carcasses (Sebastián‐González et al. 2019). Continued research across varied geographical and ecological contexts using a combination of these metrics is necessary for planning effective conservation and management actions to protect the ecosystem services that scavengers provide.

Apex carnivores exert well‐known keystone effects, including maintaining balanced ecosystems through direct and indirect effects on prey populations (Estes et al. 2011) and providing carrion (i.e., energy and nutrients) to scavengers (Wilmers et al. 2003; Allen et al. 2015; Panda et al. 2023). When feeding on carrion derived from kills made by apex carnivores, scavengers are also at risk of injury or death, and thus may alter their spatiotemporal behavioral patterns to reduce their exposure to risk posed by more dominant carnivores (i.e., landscape of fear; Wilmers et al. 2003; Prugh and Sivy 2020; Panda et al. 2023). These effects can be species‐specific, as interspecific competition is often strongest among species with overlapping niches (Pianka 1974), and larger mesocarnivores scavenge more frequently from ungulate carcasses (Prugh and Sivy 2020). Variation in the size of prey the apex carnivore kills can also affect the speed of discovery and quantity of carrion available (Inagaki et al. 2022). Collectively, these individual‐ and species‐level interactions highlight the role of apex carnivores in structuring scavenger communities and food webs.

Pumas ( *Puma concolor* ) are solitary, wide‐ranging apex carnivores that primarily prey on large ungulate prey and have outsized and irreplaceable roles in creating food web links and stabilizing ecosystems (LaBarge et al. 2022). When pumas kill large prey, they frequently leave behind partially consumed remains that provide substantial food resources for a diversity of scavengers (Allen et al. 2015, 2026; Elbroch, O'Malley, et al. 2017; Perrig et al. 2017). Scavengers that have been documented feeding on carrion from puma kills include large competitors like bears (*Ursus* spp.), mesocarnivores including coyotes ( *Canis latrans* ), and numerous avian species from Andean condors ( *Vultur gryphus* ) and vultures to songbirds (Allen et al. 2015; Perrig et al. 2017). Pumas can experience substantial resource losses to dominant scavengers (i.e., American black bears, *Ursus americanus* ; Allen et al. 2021) and human disturbance (Smith et al. 2017; Allen et al. 2026), both of which can cause pumas to abandon kills, reduce feeding times, and increase the intervals between feeding visits. Understanding the functional ecological relationships between pumas and scavengers is therefore critical for developing effective conservation strategies and understanding local food webs.

While the presence of the apex carnivore that provisioned the carrion is a key factor affecting scavenger activity, there are additional factors (interspecific competition with other scavengers, traits, seasons, and other environmental changes) that also affect scavengers at kills. Because carrion is an ephemeral resource, there can be frequent and intense interspecific competition among scavengers, which can affect the duration of feeding and the amount of nutrition acquired (Allen et al. 2016; Sebastián‐González et al. 2016; Inagaki et al. 2023). This competition can vary seasonally with changing abundance of species and environmental conditions, as can scavenging behavior overall—with some species being better able to exploit carrion during specific times of the year (Sebastián‐González et al. 2019; De Pelsmaeker et al. 2024). The ability of scavengers to find and exploit carrion resources is also dependent on species‐specific traits (including advanced olfactory capabilities, fighting ability, and other behavioral adaptations) (Allen et al. 2016; Naves‐Alegre et al. 2022; Sebastián‐González et al. 2021). These traits are particularly important for early detection of carcasses, with the ephemeral nature of carrion often leading to scramble competition for the resultant resource (Inagaki et al. 2022). Better understanding these complex factors that affect the ability of scavengers to exploit carrion resources is essential for comprehending the ecological roles of scavengers and their impact on ecosystem dynamics.

We investigated the scavenger assemblages at carcasses of ungulates recently killed by pumas (*n* = 62) in the Santa Cruz Mountains of California using video camera traps. For each scavenger species, we documented the number of kills visited, the maximum number of individuals visiting simultaneously, the number of visits to each kill, mean visit duration, and total time spent at each kill. We hypothesized that in this human‐dominated landscape obligate avian scavengers (i.e., turkey vultures, *Cathartes aura* ) would be the main scavenger (defined as feeding on more carcasses than other scavengers; Inagaki, Allen, Sebastián‐González, and Koike 2025) and visit more kills than opportunistic scavengers (e.g., coyotes) (Sebastián‐González et al. 2019). We also predicted that larger scavengers would spend longer at kills than smaller scavengers due to being the dominant scavenger (defined as competitively dominant species; Inagaki, Allen, Sebastián‐González, and Koike 2025) and having reduced fear of competition (Prugh and Sivy 2020). We also predicted that scavenging would vary by season (Sebastián‐González et al. 2019; De Pelsmaeker et al. 2024), hypothesizing that scavenging activity would be lower for species in the dry season because it is hotter and thus has greater decomposer activity and competition from them (Ray et al. 2014). Finally, because the speed of carcass discovery can affect a scavenger's ability to find and exploit ephemeral carrion resources (Inagaki et al. 2022), we predicted that scavenger species that first discovered carcasses were more likely to visit a greater number of carcasses, and that scavengers with shorter mean detection times would be associated with greater total time spent at carcasses.

## 2 Materials and Methods

### 2.1 Study Area

**FIGURE 1:**
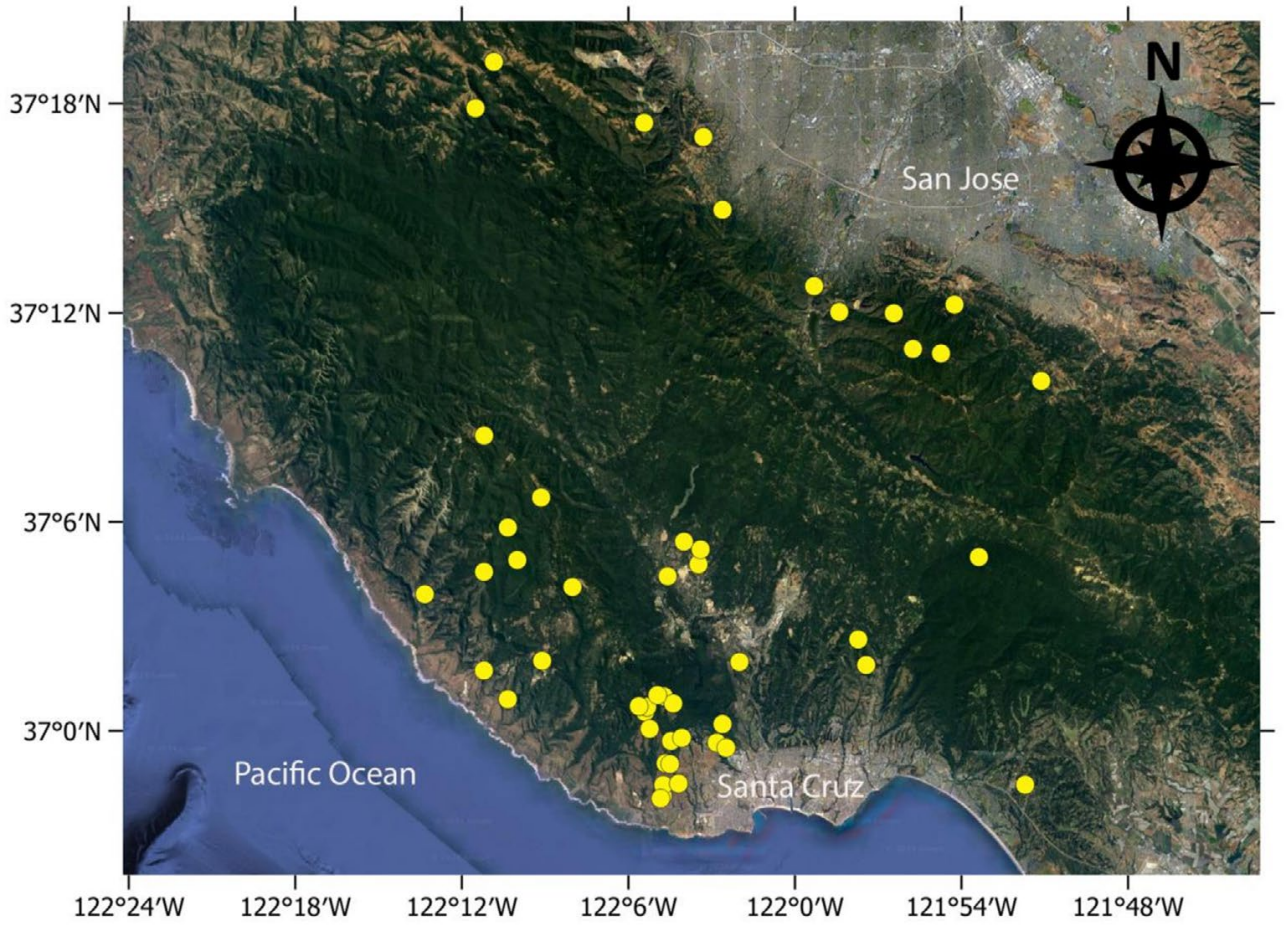
The study area in the Santa Cruz Mountains of California, with each monitored ungulate killed by a puma noted as a yellow dot.

We studied the activity of scavengers at puma kills in the Santa Cruz Mountains, California, USA (37° 10′ N, 122° 3′ W) from 2018 through 2024. We used an effective study area of approximately 2000 km^2^, with elevations ranging from sea level to 1155 m. The Pacific Ocean was the boundary to the west and south, with the cities of Palo Alto and San Jose the effective boundaries to the north and east (Figure 1). The Santa Cruz region supports a mosaic of diverse habitats from wild to highly developed areas (e.g., Silicon Valley) (Yovovich et al. 2020). There are two distinct seasons: the wet season from November to April, with daily temperature highs typically of 15.5°C–18°C when nearly all of 58–121 cm of rainfall occurs, and the dry warmer season with daily temperature highs of 18°C–26°C and sparse to no rainfall.

### 2.2 Field Methods

We followed the ethical guidelines of the American Society of Mammalogists (Sikes et al. 2011) and procedures approved by the Independent Animal Care and Use Committee at the University of California, Santa Cruz (Protocols Wilmc0709 and Wilmc1101) for animal captures. From 2008 to 2024, we captured 145 pumas using trained hounds or box traps. Upon capture, we anesthetized the animals (primarily using Telazol; tiletamine HCl and zolazepam HCl; Fort Dodge Animal Health, Fort Dodge, IA) and assessed their health. We fit each with an individually identifiable ear tag and a satellite‐enabled GPS collar (Vectronics Aerospace, Berlin, Germany). We generally programmed collars to take a GPS point at 4‐h intervals and download the data daily via satellite.

To monitor scavenger activity at puma kills, we examined GPS data from puma collars to identify freshly killed ungulates from 2018 to 2024. We visited potential kills at recently formed clusters (with at least one nocturnal point and at least two GPS points within 100 m of each other; Elbroch and Wittmer 2012; Allen et al. 2026). When we were successful in finding a freshly killed ungulate at the points, we secured the carcass with a rope to a tree or earth anchor to keep it within the viewshed of the camera. We then set a video camera trap (Reconyx Hyperfire, Holmen, WI) on a nearby tree to monitor puma and scavenger activity at the kill. We programmed the camera traps to take 1‐min videos with a 1‐s delay between videos. We kept the camera traps at the kill until it was completely consumed, typically for a minimum of 3 weeks.

In our analyses of scavenger activity at puma kills, we excluded kills where we did not record the puma feeding at the kill (*n* = 12). We therefore ended up using 50 kills, including black‐tailed deer (*n* = 49, *Odocoileus hemionus* ) and wild boar (*n* = 1; *Sus scrofa* ). After collecting the camera, we trimmed the data to 21 days from the time of the kill (the initial GPS point of the kill cluster) for our analyses to standardize the data and because nearly all scavenging typically occurs within 3 weeks (Inagaki et al. 2022). We only considered the activity of scavengers that directly interacted with the carcass (feeding, investigating, scent marking, or other behaviors) in our analyses. We labeled up to a maximum of 5 individuals feeding concurrently, due to the difficulty of accuracy at greater numbers; in cases with > 5 individuals, we considered there to be 6 individuals in our analyses. We reviewed each video, noting each behavior that pumas and scavengers exhibited. We defined a visit as the continuous time an animal was in the viewshed of the camera interacting with the carcass.

### 2.3 Statistical Analyses

We used R (version 4.4.1; R Core Team 2024) for our statistical analyses, and considered *p* ≤ 0.05 to be statistically significant. We first calculated summary statistics for each scavenger species, including (a) the number of kills each scavenger species visited, (b) the number of kills at which each scavenger species was the first species to arrive, (c) the mean time for each scavenger species to first detect carcasses (calculated from the estimated time of the kill from the GPS collar), the mean number of visits that each scavenger species made to kills, the mean visit durations in minutes for each scavenger species at kills, and the total time in minutes each scavenger species spent at kills. To understand overall patterns of scavenging between seasons, we used a series of *t*‐tests between wet and dry seasons for detection time, mean visit duration, and total time at kills for each scavenger species. We later used linear regression to compare the number of kills visited with the number of kills visited first, and to compare detection time with total time spent at kills.

## 3 Results

At the puma‐killed ungulate carcasses that we monitored (*n* = 50), we documented 485 visits by the pumas that made the kill, lasting a total of 11,950 min. We also documented a total of 14 scavenger species (including scavenging pumas), for which we documented 819 visits for a total of 7813 min (Table 1).

**TABLE 1:** Scavenger community metrics of each scavenger species at carcasses of 50 ungulates killed by pumas ( *Puma concolor* ) in the Santa Cruz Mountains, California. Metrics include the number of kills visited, mean maximum individuals documented at kills (range), mean number of visits (range), mean visit duration in minutes (±standard deviation), and total time in minutes (±standard deviation, range).

Scavenger species	Number of kills visited	Maximum individuals	Number of visits	Mean visit duration	Total time
Bobcat	11	1.00 (1–1)	4.0 (1–15)	8.87 (±5.34)	22.45 (±9.82; 1–102)
Common raven	12	1.67 (1–3)	6.0 (1–18)	5.83 (±2.33)	38.58 (±13.73; 1–152)
Coyote	7	2.57 (1–5)	3.4 (1–8)	7.47 (±2.07)	28.43 (±9.89; 1–71)
Domestic dog	4	1.25 (1–2)	2.7 (1–7)	5.91 (±3.76)	12.50 (±6.55; 1–29)
Gray fox	6	1.00 (1–1)	3.5 (1–14)	6.41 (±4.16)	19.17 (±12.74; 1–80)
*Peromyscus* sp.	11	1.09 (1–2)	5.4 (1–27)	1.18 (±0.15)	9.82 (±6.42; 1–73)
Opossum	17	1.06 (1–2)	10.2 (1–41)	4.28 (±0.71)	46.65 (±12.14; 1–158)
Puma	10	1.20 (1–2)	2.0 (1–8)	19.75 (±8.01)	66.40 (±44.46; 1–456)
Red‐tailed hawk	5	1.20 (1–2)	3.4 (1–8)	4.87 (±2.51)	16.40 (±8.20; 1–40)
Scrub jay	1	1.00 (1–1)	1.0 (1–1)	1.00 (±0.00)	1.00 (±0.00; 1–1)
Steller's jay	2	2.50 (2–3)	26.0 (23–29)	3.39 (±2.08)	82.00 (±44.00; 38–126)
Striped skunk	5	1.00 (1–1)	1.6 (1–3)	1.00 (±0.00)	1.60 (±0.40; 1–3)
Turkey vulture	22	2.45 (1–6)	11.1 (1–47)	15.75 (±4.08)	212.23 (±68.95; 1–1234)
Dusky‐footed woodrat	10	1.10 (1–2)	7.3 (1–32)	1.58 (±0.39)	25.00 (±15.68; 1–123)

On average, individual scavenger species spent 63.52 (±14.42 SD, range = 1–1234) min at puma kills, with substantial variation among species. Turkey vultures spent the greatest average total time at kills (212.3 min, Table 1), followed by Steller's jays ( *Cyanocitta stelleri* ; 82.0 min) and scavenging pumas (66.4 min). Several species spent relatively little time at kills (averaging < 15 total min at kills), including domestic dogs ( *Canis familiaris* ), mouse sp., striped skunks ( *Mephitis mephitis* ), and scrub jays ( *Aphelocoma californica* ) (Table 1). The only species that exhibited seasonal variation was turkey vultures, which spent more time at kills in the dry season ($\bar{x}$ = 372.70 ± 132.99 SE) than in the wet season ($\bar{x}$ = 78.50 ± 31.70 SE; df = 20, *t* = 2.34, *p* = 0.029).

On average, individual scavengers had mean visit durations of 7.84 (±1.23 SE, range = 1.00–71.40) min at puma kills, and also varied by species. Scavenging pumas had the greatest mean visit duration (19.8 min, Table 1), followed by turkey vultures (15.8 min), bobcats ( *Lynx rufus* ; 8.9 min), and coyotes (7.5 min). Multiple species also averaged less than 2 min at kills, including mice, California scrub jays, striped skunks, and dusky‐footed woodrats ( *Neotoma fuscipes* ) (Table 1). None of the scavenger species varied between seasons in their mean visit duration.

Coyotes were the most gregarious scavengers, with an average of 2.57 individuals present at kills (maximum = 5, Table 1), followed by Steller's jays ($\bar{x}$ = 2.45, maximum = 3) and turkey vultures ($\bar{x}$ = 2.45, maximum > 5). Three species were documented only as lone individuals at kills, including bobcats, gray foxes ( *Urocyon cinereoargenteus* ), and California scrub jays (Table 1).

**FIGURE 2:**
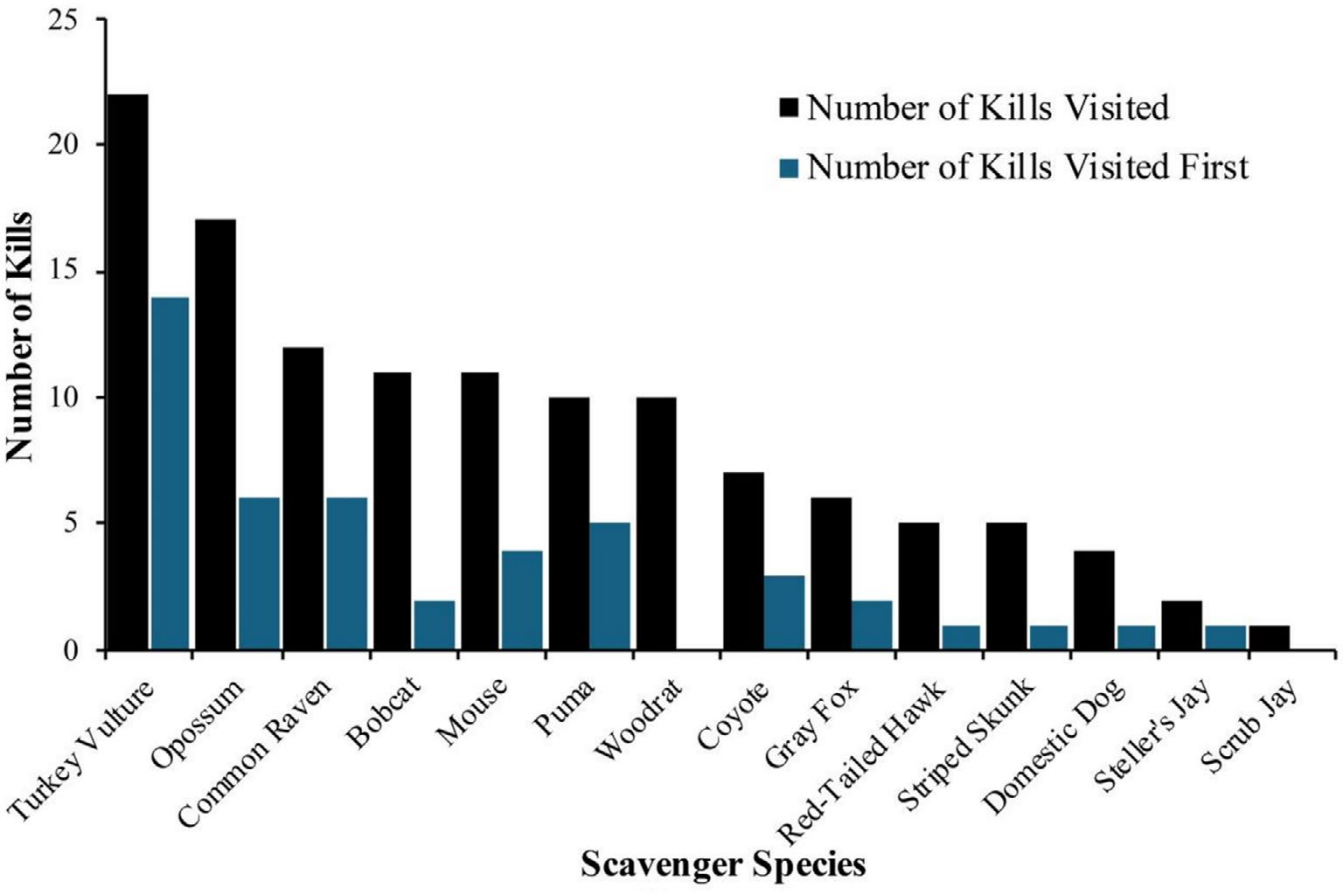
The count of number of kills visited for each scavenger species and the number of kills where the species was the first to discover the carcass.

**FIGURE 3:**
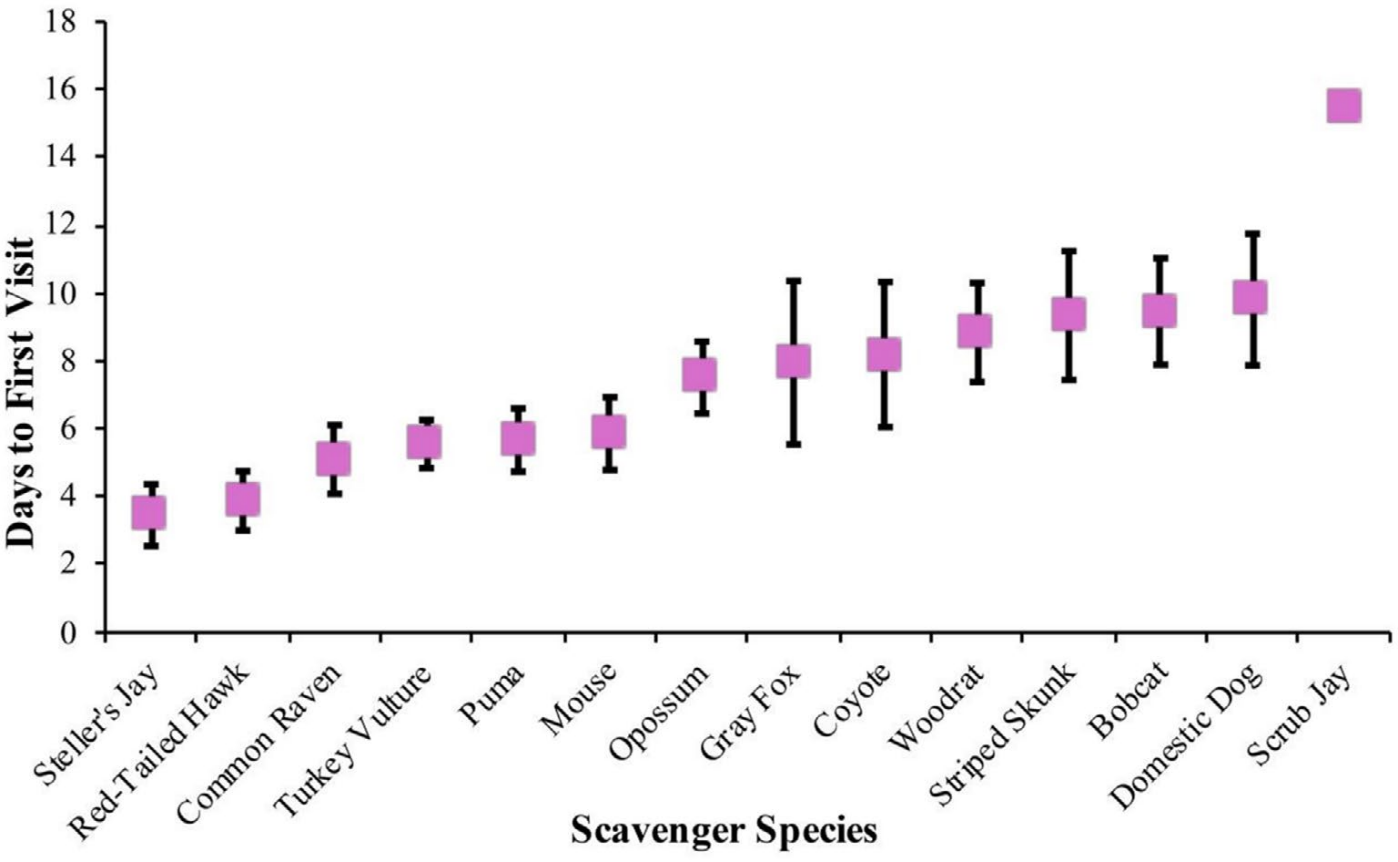
The mean times to detect carcasses for each scavenger species documented, with the error bars representing the standard deviation.

On average, scavengers first arrived at kills 6.97 (±0.39 SD, range = 1.11–17.52) days after the puma made the kill, but this varied by species. Steller's jays detected carcasses most quickly (3.46 days, Figure 3), followed by red‐tailed hawks ( *Buteo jamaicensis* ; 3.88 days), common ravens (*Corvus corvax*, 5.10 days), and turkey vultures (5.55 days). There was a significant but weak correlation between the speed of carcass detection and total time spent at carcasses (df = 122, *R* ^2^ = 0.07, *p* = 0.004, Figure 2). The only species that showed seasonal variation was turkey vultures, which found kills more quickly in the dry season ($\bar{x}$ = 3.35 ± 0.60 SE) than in the wet season ($\bar{x}$ = 7.39 ± 0.92 SE; df = 20, *t* = 3.51, *p* = 0.002).

Overall, the first scavenger to arrive at the carcasses arrived in 4.65 (±2.99 SD, range = 1.11–14.72) days after the puma made the kill. Turkey vultures visited the most kills (*n* = 22, Table 1) and were also the first scavengers at the most kills (*n* = 14, Figure 2). Virginia opossums (*Didelphus virginianus*; *n* = 17) visited the second most kills (*n* = 17) and were tied with common ravens for being the first scavenger at the second most kills (*n* = 6; Figure 2). There was a strong and significant correlation between the number of kills a species visited and the number of kills the species was first to arrive at (df = 13, *R* ^2^ = 0.76, *p* < 0.0001, Figure 2).

## 4 Discussion

Our findings further document the diverse scavenger assemblages that can be found at the kills of apex carnivores. At puma kills in the Santa Cruz Mountains, we documented 14 species that varied in their use of carrion, often in alignment with our predictions. Turkey vultures were the main scavengers, found kills most quickly, and spent the most time at kills in our system. It has been observed that pumas can limit the scavenging of turkey vultures and other scavengers at their kills through caching or moving carcasses into thicker cover (Allen et al. 2023); however, these concealment strategies may not have been effective for limiting discovery by turkey vultures and other avian scavengers in the Santa Cruz Mountains. This prevalence of obligate avian scavengers is similar to the Andes, where condors were highly efficient at detecting and consuming a large amount of carrion from puma kills (Perrig et al. 2017). However, this prevalence of avian scavengers varies from systems where avian scavengers are largely absent, such as Japan, where mammals are often the first to discover carcasses and consume most of the carrion (Inagaki et al. 2022). Among mammalian scavengers, Virginia opossums visited the most carcasses and spent the most time at kills, but larger scavengers (pumas, coyotes, and bobcats) had longer mean visit durations and generally spent a comparable amount of time at kills. As a smaller mesopredator, Virginia opossums may be more abundant in the system (making them more likely to find kills), but they have also been documented as the most prevalent scavengers in other areas (Olson et al. 2016), and they may be an important scavenger in North America.

Larger mesocarnivores tend to consume more carrion than other mammals, especially from ungulate carcasses (Prugh and Sivy 2020), but are also often injured or killed through competition with apex carnivores (Donadio and Buskirk 2006; Prugh and Sivy 2020). Despite the risk of injury or death, pumas' two closest competitors in Santa Cruz (coyotes and bobcats) fed for long mean visit durations (the longest except for turkey vultures and scavenging pumas) at the puma kills. Despite their similar mean feeding durations, the composition of bobcat and coyote groups while scavenging differed: coyotes often scavenged in pairs or small groups, whereas bobcats were exclusively solitary. Also, although coyotes and bobcats were observed at the same kill on three occasions, they never occurred synchronously. Coyotes and bobcats are direct competitors that rely on ephemeral carrion as a resource, and their interactions at carcasses may be shaped by individual traits (sex and age) that make some individuals more competitively dominant (Allen et al. 2016). Further research is therefore needed to understand how individual and species‐level behavioral traits (e.g., risk taking and human tolerance, see LaBarge et al. 2024) affect interspecific dynamics at carrion derived from apex carnivores.

The other scavenger species of importance was pumas themselves (observed at 20% of puma kills). Despite this relatively high rate of scavenging, we only documented agonistic encounters at one of the kills. While previous studies have noted the scavenging behavior of pumas (Bauer et al. 2005; Knopff et al. 2010), it was often carrion derived from anthropogenic sources. More recent research and the high rate of scavenging by other pumas at puma kills that we documented highlight the potential for prey sharing among pumas in the system (e.g., Elbroch, Levy, et al. 2017). Scavenging pumas had the longest mean feeding durations at the carcasses, suggesting that their presence may add to the original fear effects imposed by the puma responsible for the kill, creating longer‐lasting stimuli that prey and competitor species may learn to avoid (Laundré et al. 2010).

Comparing this study to others highlights that the composition of scavenger assemblages and main scavengers at carrion provided by pumas varies widely across ecosystems, likely influenced by differences in community structure, climate, and competition. In South America, the main scavenger was typically avian (Andean condors; Elbroch and Wittmer 2012; Perrig et al. 2017), while in North America, they were typically mammalian (American black bears in California; Allen et al. 2015; red foxes [ *Vulpes vulpes* ] in Wyoming; Elbroch, O'Malley, et al. 2017). Variation in scavenger assemblages likely affects how individual species scavenge (e.g., the lack of dominant scavengers, such as American black bears and Andean condors, likely increases scavenging opportunities for others). These differences among ecosystems suggest that scavenger assemblage structure and activity are highly context‐dependent, and meta‐analyses are needed to identify broader ecological patterns and how factors such as species composition and habitat characteristics affect food webs (Sebastián‐González et al. 2021).

Detecting a carcass early is often linked to a scavenger's ability to exploit carrion, but early arrival does not necessarily translate to prolonged feeding or greater total energy gain (Inagaki et al. 2022). Our results showed a significant but weak correlation for quicker detection leading to more time at carcasses, but a strong and significant correlation between the number of kills a species visited and the number of kills in which they were the first scavenger to arrive. This supported our prediction and shows that species that are more efficient at locating carcasses tend to visit more kills overall. Some scavenger species are known to signal the presence of carcasses to others (Naves‐Alegre et al. 2022), with obligate avian scavengers often directly tied to mammalian scavenging rates (Ogada et al. 2012). Turkey vultures, the only obligate scavenger in the system, likely fill this role as the signaler of carrion in the Santa Cruz Mountains, as they were typically the first scavenger to arrive at kills and visited the most kills. However, all four species to discover carcasses most quickly (on average) were avian scavengers, and it may be that avian scavengers generally act as signalers of the presence and location of a carcass.

Scavenger species varied in their use of puma kills, but despite distinct seasons, we did not detect many seasonal differences in scavenging activity. The only species with significant seasonal variation in response to carcasses was turkey vultures, which found kills more quickly and spent more than three times the duration at kills in the dry season. We expected scavenging activity to be lower in the dry season because it is hotter, which leads to greater competition with decomposers and a more rapid reduction in the palatability of food (Ray et al. 2014), but found the opposite effect. Human activity may be greater in the dry season, which may make larger mammalian scavengers wary and less likely to spend time at kills. This could provide greater opportunities for avian scavengers, or there could be changes in the microhabitat of where kills occur in different seasons (with varying cover).

Our study highlights the complex dynamics of scavenger interactions at puma kills in the Santa Cruz Mountains and how they vary in some respects from scavenger assemblages in different ecosystems. We found that turkey vultures were the main scavenger species in our system, as is typical of systems where obligate scavengers are present (Ogada et al. 2012; Naves‐Alegre et al. 2022). But large scavengers (bobcats and coyotes), as well as other pumas and opossums, were also important. This emphasizes the importance of species‐specific traits and ecological context in shaping scavenger communities. Our findings contribute to a broader understanding of scavenger ecology, specifically at puma kills, which is important due to the role of pumas as ecological brokers (LaBarge et al. 2022). Given the variation observed across different systems, meta‐analyses are needed to assess broader patterns in scavenger dynamics and their implications for food webs. Future research should further explore the behavioral and ecological factors driving the formation of scavenger assemblages to inform conservation and management strategies for ecosystems where apex carnivores provide critical carrion resources.

## Author Contributions

**Maximilian L. Allen:** conceptualization (lead), data curation (lead), formal analysis (equal), funding acquisition (supporting), investigation (lead), methodology (lead), visualization (lead), writing – original draft (lead). **Andrew T. L. Allan:** conceptualization (supporting), methodology (supporting), writing – original draft (supporting), writing – review and editing (equal). **Richard M. King:** data curation (supporting), investigation (lead), writing – review and editing (equal). **Bethany H. Warner:** data curation (equal), writing – review and editing (equal). **John J. Morgan:** data curation (supporting), investigation (supporting), writing – review and editing (equal). **Christopher C. Wilmers:** conceptualization (supporting), funding acquisition (lead), methodology (supporting), writing – review and editing (equal).

## Funding

Christopher C. Wilmers received funding for this work from National Science Foundation grants #0963022 and #1255913, and the Gordon and Betty Moore Foundation. M. Allen received funding for this work from the University of Illinois, Prairie Research Institute, and the Illinois Natural History Survey. Andrew T. L. Allan received funding for this work from the Leverhulme Trust (ECF‐2023‐318).

## Conflicts of Interest

The authors declare no conflicts of interest.

## Data Availability

The data for the manuscript is archived in the Illinois Data Bank: https://doi.org/10.13012/B2IDB‐3246094_V1
